# Epidemiological Surveillance of Low Pathogenic Avian Influenza Virus (LPAIV) from Poultry in Guangxi Province, Southern China

**DOI:** 10.1371/journal.pone.0077132

**Published:** 2013-10-30

**Authors:** Yi Peng, Zhi-xun Xie, Jia-bo Liu, Yao-shan Pang, Xian-wen Deng, Zhi-qin Xie, Li-ji Xie, Qing Fan, Si-si Luo

**Affiliations:** Guangxi Key Laboratory of Animal Vaccines and Diagnostics, Guangxi Veterinary Research Institute, Nanning, Guangxi Province, China; National Center for Biotechnology Information (NCBI), United States of America

## Abstract

Low pathogenic avian influenza virus (LPAIV) usually causes mild disease or asymptomatic infection in poultry. However, some LPAIV strains can be transmitted to humans and cause severe infection. Genetic rearrangement and recombination of even low pathogenic influenza may generate a novel virus with increased virulence, posing a substantial risk to public health. Southern China is regarded as the world “influenza epicenter”, due to a rash of outbreaks of influenza in recent years. In this study, we conducted an epidemiological survey of LPAIV at different live bird markets (LBMs) in Guangxi province, Southern China. From January 2009 to December 2011, we collected 3,121 cotton swab samples of larynx, trachea and cloaca from the poultry at LBMs in Guangxi. Virus isolation, hemagglutination inhibition (HI) assay, and RT-PCR were used to detect and subtype LPAIV in the collected samples. Of the 3,121 samples, 336 samples (10.8%) were LPAIV positive, including 54 (1.7%) in chicken and 282 (9.1%) in duck. The identified LPAIV were H3N1, H3N2, H6N1, H6N2, H6N5, H6N6, H6N8, and H9N2, which are combinations of seven HA subtypes (H1, H3, H4, H6, H9, H10 and H11) and five NA subtypes (N1, N2, N5, N6 and N8). The H3 and H9 subtypes are predominant in the identified LPAIVs. Among the 336 cases, 29 types of mixed infection of different HA subtypes were identified in 87 of the cases (25.9%). The mixed infections may provide opportunities for genetic recombination. Our results suggest that the LPAIV epidemiology in poultry in the Guangxi province in southern China is complicated and highlights the need for further epidemiological and genetic studies of LPAIV in this area.

## Introduction

Avian influenza (AI) is caused by specified avian influenza viruses (AIVs) that belong to the genus Influenza virus A, of the family Orthomyxoviridae. In terms of antigen variations of the surface glycoprotein hemagglutinin (HA) and neuraminidase (NA), AIVs are further divided into different subtypes. So far, 16 HA subtypes and 9 NA subtypes of AIVs have been identified and described [1]–[3]. Among these subtypes, H5N1 and H7N2 cause high pathogenic avian influenza (HPAI), which is characterized by systemic infections, high mortality and morbidity. Low Pathogenic Avian Influenza (LPAI) can be caused by all HA subtypes (H1–H16) [4], [5]. Poultry infected with LPAI usually have mild symptoms or are asymptomatic carriers, but it is possible to transmit certain viruses to humans, which are capable of causing severe illness. For example, H7N9 are a LPAIV that has been the cause of worldwide outbreaks of avian influenza.

It has been confirmed that genetic recombination in LPAIV has led to deleterious gene mutations, novel phenotypes and increased virulence [6]. Previous studies have shown that seven genes of the H5N1 virus isolated in the Hong Kong outbreak in 1997 have high sequence similarity to the LPAIV virus H6N1 [7]. Some LPAIV, such as H9N2, can break species barriers and provide genes to other influenza virus, which could present a risk for severe human infection. It has been suggested that eight of the genes from the H1N1 subtype that caused the 1918 pandemic were originally from a poultry virus [8]. It has also been suggested that this outbreak was transmitted to humans via a pig intermediary [9], [10]. The HA and PB1 genes of the H3N2 subtype that caused the outbreak in Hong Kong in 1968 were originally from the H3 subtype of AIV [11], [12]. Since 1998, the H9N2 subtype has caused several cases of human infections in mainland of China and Hong Kong. These subtypes posed great threats to public health [13], [14]. Thus, epidemiological surveys of HA subtypes of LPAIV in Southern China are critically important.

Southern China is regarded as an “influenza epicenter”, due to the recent influenza outbreaks in Hong Kong and Southern Asian [15], [16]. This area is on the migratory path of many bird species and is highly populated with poultry, animals and humans. There are several large-scale live bird markets (LBMs) in this area and a large number of small-scale poultry farms. Close contacts between the poultry, animals and humans may facilitate transmission of the influenza virus. In addition, the warm, humid climate in this region may prompt long-term survival and proliferation of the virus. Furthermore, the local people usually use poultry manure to feed their pigs and then the pig manure is used to feed fish. These activities may facilitate the transmission of influenza virus among animals, birds and poultry via water, feed, feces and urine, providing opportunities for genetic recombination of the influenza virus. Recently, H7N9 influenza viruses have caused severe infection in humans in China and transmission by respiratory droplets has been shown in ferrets [17], [18]. In this study, we conducted epidemiological surveillance of LPAIV at LBMs in Guangxi province in southern China. The epidemiological data from this region will be useful to plan strategies for preventing LPAIV from causing human infections. Additionally, the results from this surveillance may be helpful in influencing worldwide surveillance of LPAIV, given that this region is on the route of bird immigration.

## Materials and Methods

### Ethics statement

This study was approved by the Animal Ethics Committee of the Guangxi Veterinary Research Institute, which supervises all live bird markets (LBMs) in Guangxi province. The institute did not issue a number or ID to this animal study, because the studied birds are not an endangered or protected species and birds were not sacrificed for sampling. Sample collection was conducted based on the protocol issued by the Animal Ethics Committee of the Guangxi Veterinary Research Institute. Briefly, with verbal permission from the bird owners, biological samples were gently collected from the cloaca, larynx and trachea of healthy chickens and ducks, using sterilized cotton swabs. The birds were not anesthetized before sampling and the sampled birds were observed for 30 mins after sampling, before being returned to their cages.

### Field work

The epidemiological surveillance of LPAIV in poultry was conducted in 15 randomly selected LBMs in the Guangxi province of southern China during the period from January 2009 to December 2011. Biological samples were collected from the cloaca, larynx and trachea of healthy chickens and ducks using cotton swabs. The cotton swabs were then suspended in 1ml of storage medium that was transported in 1.5 ml finger tubes on ice. According to the protocol of the world organization for animal health (OIE), the storage medium contained Penicillin (10,000 unit/ml), Streptomycin (10 mg/ml), Gentamycin (10,000 unit/ml), Kanamycin (10000 Unit/ml) and 5% fetal calf serum in sterile PBS (pH  = 7.2).

### Isolation of virus

The samples were centrifuged at 3000 rpm for 10 min at 4°C, while still in the storage medium. Supernatants were collected and stored at −70°C for virus isolation. The Specific pathogen-free (SPF) eggs were purchased from the Bejing Merial Biology Company (Beijing, China). SPF chicken embryos (9–10 days of age) were used for virus inoculation. Each sample (supernatant) was inoculated into three SPF chicken embryos (0.2 ml/embryo), via the allantoic cavity. The inoculated chicken embryos were incubated at 35°C and observed daily. The allantoic fluids were collected from the embryos that died within 120 h of inoculation. The chicken embryos that survived longer than 120 h, post inoculation, were sacrificed at 4°C and the allantoic fluids were collected. The collected allantoic fluids were then tested for hemagglutination (HA). The positive allantoic fluids were stocked at −70°C for identification of virus.

### Identification of virus

The hemagglutination inhibition (HI) assay was conducted to determine the HA subtypes of LPAIV. Briefly, the HA-positive allantoic fluids were tested with the serum of different HA subtypes, anti-Newcastle Disease virus (NDV) and anti-hemagglutinating adenovirus (EDS76) to determine the HA subtypes. The viruses with determined HA subtypes were further inoculated and grown in Madin-Darby canine kidney (MDCK) cells. The titers of viruses were determined by plaque assay.

Viral RNA was extracted using a Body Fluid Viral DNA/RNA Miniprep Kit (Axygen Biosciences, Hangzhou, China). Based on the protocol, RT-PCR assay was conducted to obtain the full length of the neuraminidase (NA) gene, using primers designed in previous studies [19]. The NA subtype was determined by further sequencing of the amplified NA genes and a BLAST search in the GenBank nr database, which contains the nucleotide sequences of all subtypes of the NA gene.

### Statistical analysis

The viral isolation rates were compared using the Fisher exact test with the Epi Info 6.0 software (Centers for Disease Control and Prevention, Atlanta, GA, USA). A difference was considered statistically significant when *p*<0.01.

## Results

### Isolation rates of LPAIV in different poultry species

**Figure 1:**
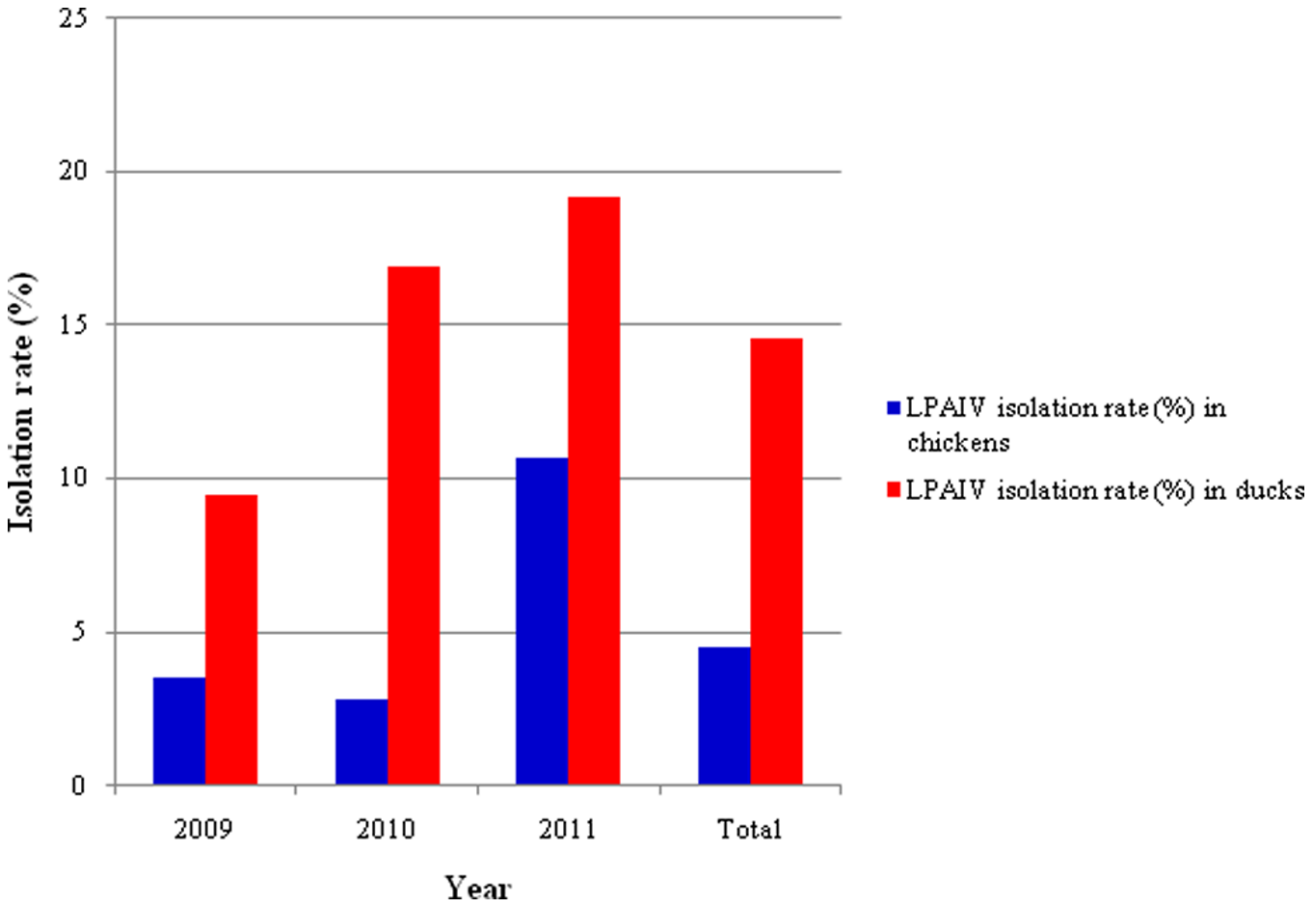
Annual isolation rates of AIV in poultry at different LBMs in Guangxi province, Southern China.

**Figure 2:**
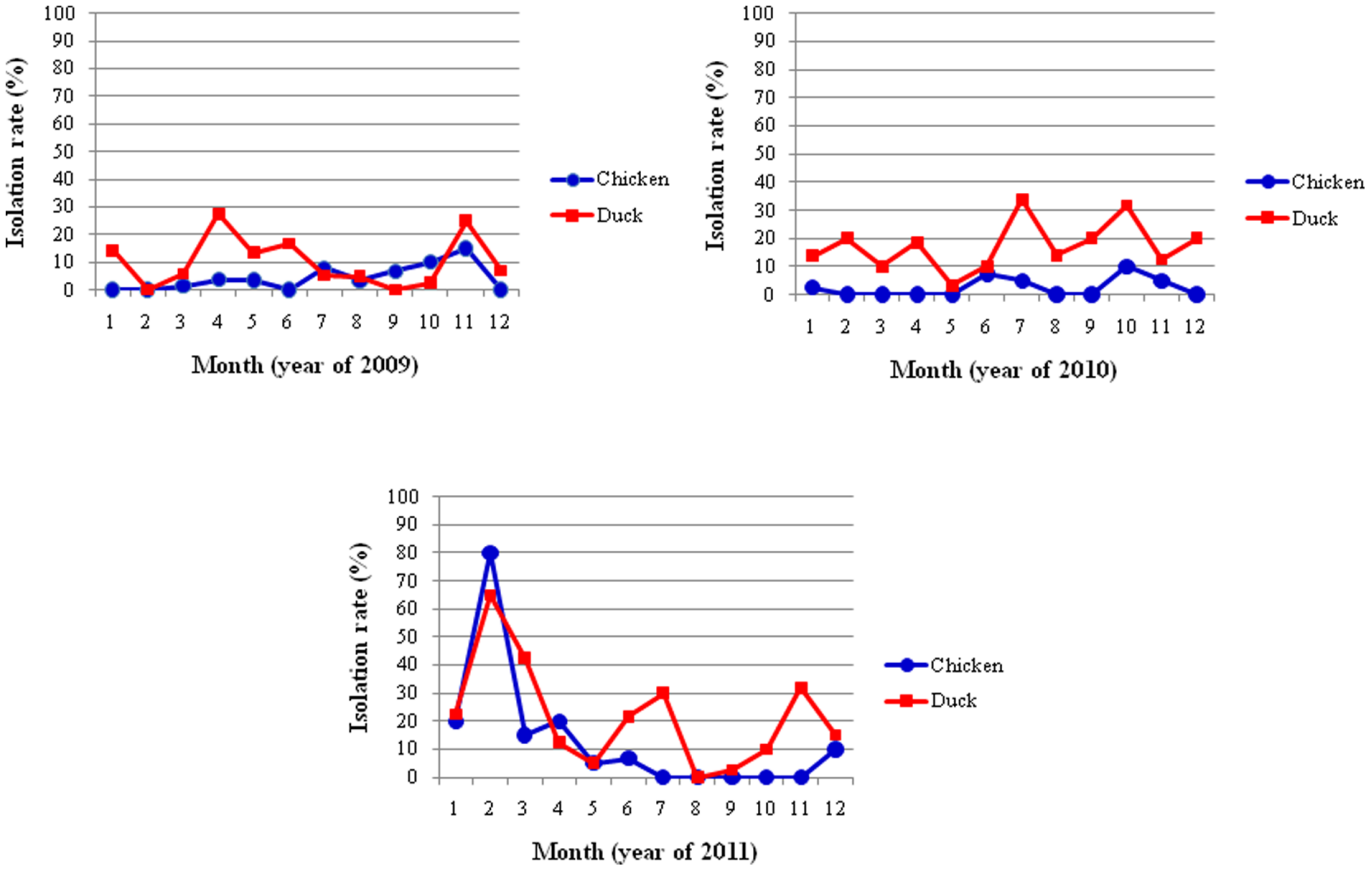
Monthly isolation rate of AIV in poultry at different LBMs in Guangxi, Southern China.

From January 2009 to December 2011, a total of 3,121 swab samples were collected from the cloaca, larynx and trachea of healthy chickens and ducks at different LBMs in the Guangxi province. Among the 3,121 samples, 336 (10.8%) were positive for LPAIV. The LPAIV isolation rate in ducks was significantly higher than in chickens (Figure 1) (*P<*0.01), which was 9.1%, whereas the LPAIV isolation rate in chickens was 1.7%. The LPAIV isolation rate in chickens in 2010 showed a slight decline, compared to the isolation rate in chickens in 2009, but increased in 2011 when compared to rates from 2009 and 2010 (Figure 1). The LPAIV isolation rate in ducks, however, showed a continual increase from 2009 to 2011 (Figure 1). In general, the seasonal pattern of the LPAIV isolation rate in ducks and chickens is highly similar (Figure 2). In February 2010, the LPAIV isolation rate reached a peak in both ducks and chickens. No significant association between the LPAIV isolation rate and season was observed, however (*P*>0.05).

### HA and NA subtypes identified in chickens and ducks

**Figure 3:**
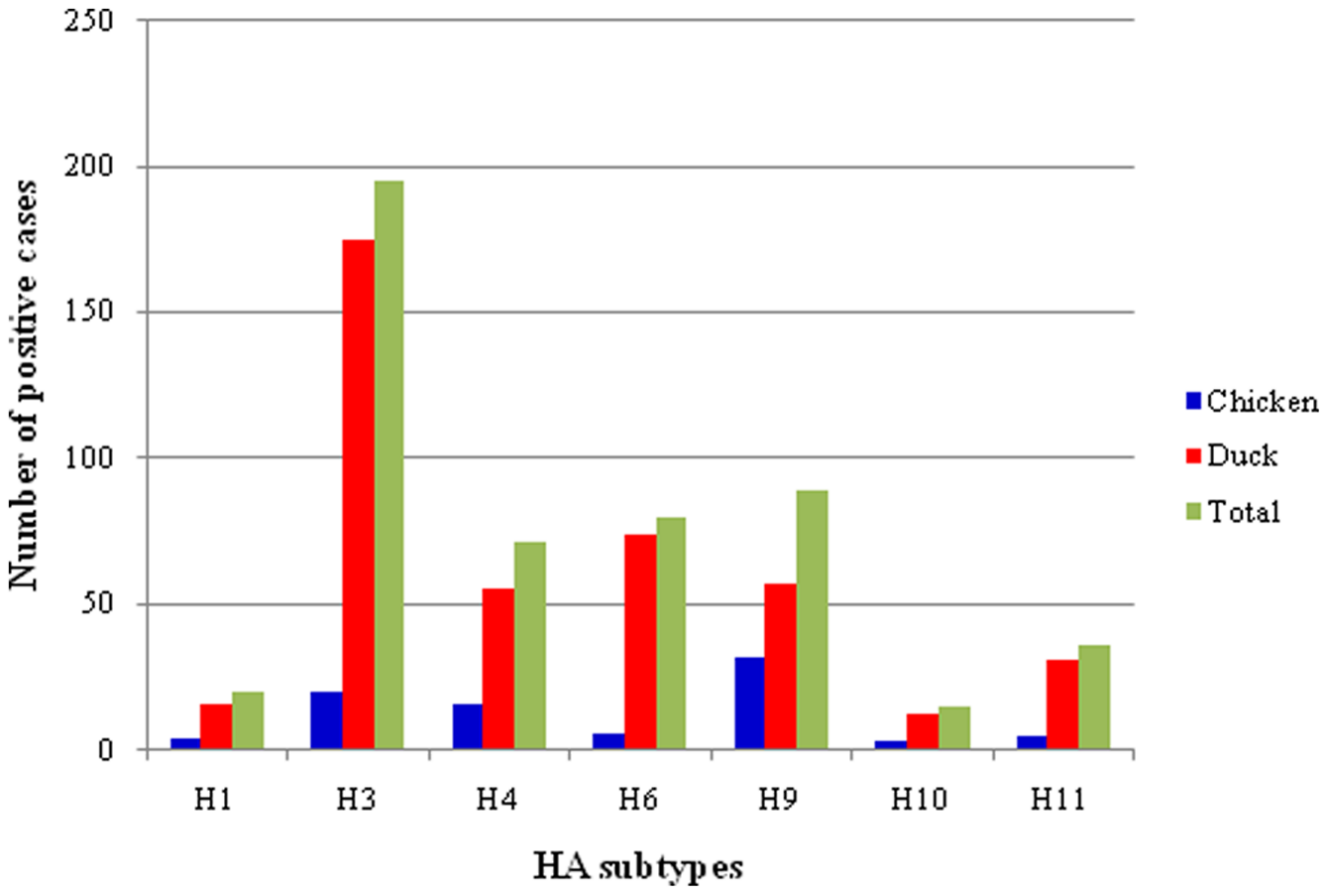
Distribution of HA subtypes and NA subtypes in poultry at different LBMs in Guangxi, Southern China.

More than seven HA subtype (H1, H3, H4, H6, H9, H10, H11 and some which were unknown) were identified in ducks and chickens (Figure 3). The H3 subtype was dominant in ducks and the H9 subtype was dominant in chickens (Figure 3). The distribution of different HA subtypes in chickens and ducks was slightly different. Based on RT-PCR, DNA sequencing and a BLAST search in the NCBI nr database, five NA subtypes, including N1, N2, N5, N6 and N8, were identified. The combination of HA and NA subtypes identified in this study are H3N1, H3N2, H6N1, H6N2, H6N5, H6N6, H6N8 and H9N2. The sequences of identified HA subtypes were submitted to GenBank (Accession numbers: JN003630, JX304754, JX297583, JX293559, JX304770, JX304762, and KC608159).

### Mixed infection of different HA subtypes

**Figure 4:**
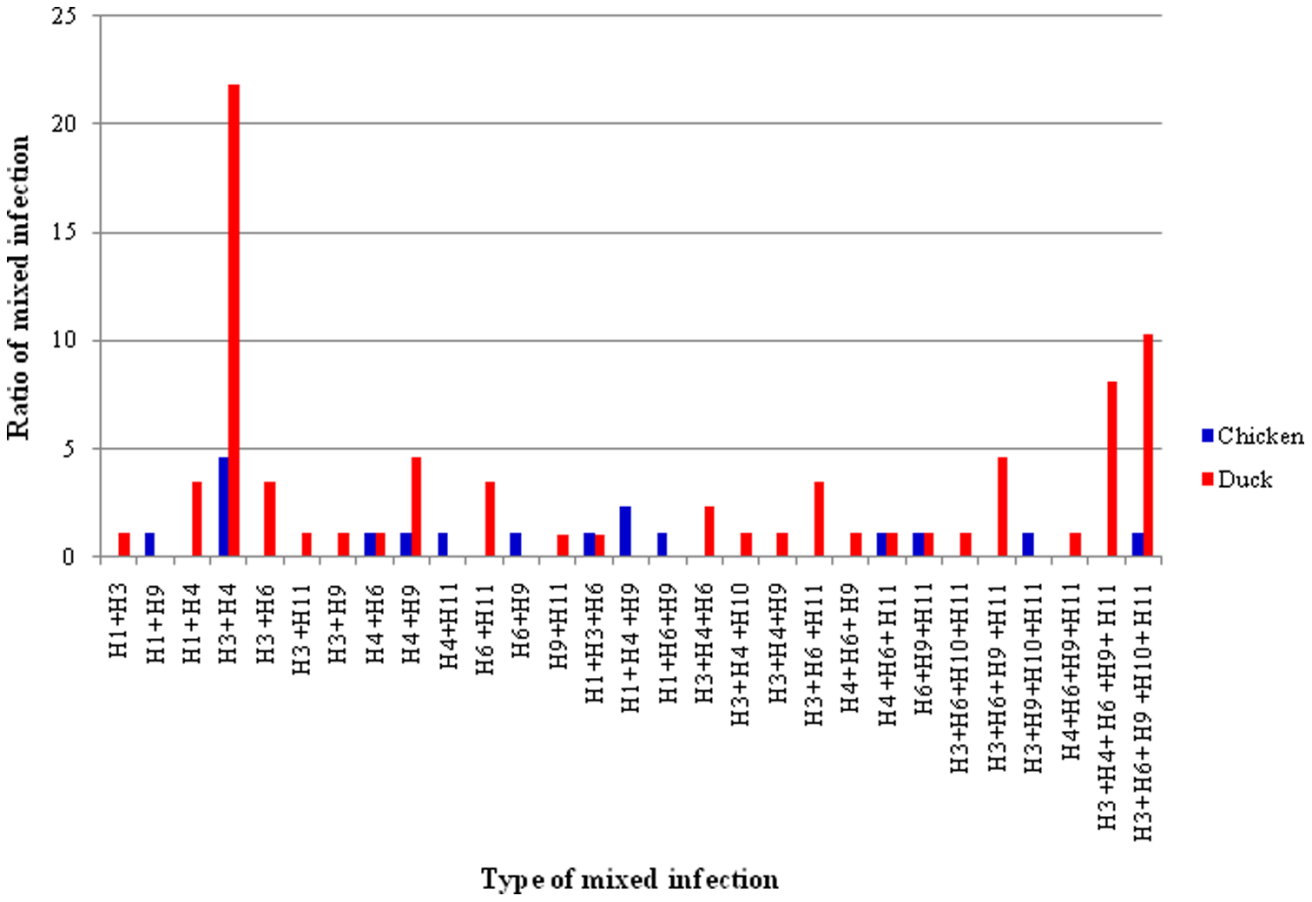
The ratio of different types of mixed infection of LPAIV in chickens and ducks.

Of the 336 samples positive for LPAIV, 88 (25.9%) (17 in chickens and 70 in ducks) showed mixed infections of different HA subtypes of LPAIV (Table 1). As shown in Tables 2, 13 and 23 types of mixed infection of HA subtypes were identified in both chickens and ducks, respectively. Among these concomitant infections, 13 were mixes of two different HA subtypes, 10 were mixes of three different HA subtypes, four were mixes of four different HA subtypes and two were mixes of five different HA subtypes (Table 2). Mixed infection of H3 and H4 subtypes was the most common mixed infection (Figure 4).

**Table 1:** LPAIV isolated from chickens and ducks in live bird markets (LBMs) in Guangxi, Southern China during the period from January 2009 to December 2011.

Date	The number of total samples		The number of LPAIV positive samples		LPAIV isolation rate (%)		The number of co-infection samples		Co-infection rate (%)*	
	Chicken	Duck	Chicken	Duck	Chicken	Duck	Chicken	Duck	Chicken	Duck
1/2009	28	14	0	2	0	14.3	0	0	0	0
2/2009	72	39	0	0	0	0	0	0	0	0
3/2009	70	51	1	3	1.4	5.9	1	2	100	66.7
4/2009	81	80	3	22	3.7	27.5	2	17	66.7	77.3
5/2009	57	52	2	7	3.5	13.5	0	3	0	42.9
6/2009	60	54	0	9	0	16.7	0	5	0	55.5
7/2009	75	57	6	3	8	5.3	0	1	0	33.3
8/2009	30	165	1	8	3.3	4.8	1	5	100	62.5
9/2009	30	78	2	0	6.7	0	0	0	0	0
10/2009	20	40	2	1	10	2.5	0	1	0	1
11/2009	20	40	3	10	15	25	3	4	100	40
12/2009	30	58	0	4	0	6.9	0	2	0	50
Total in year 2009	573	728	20	69	3.5	9.5	7	40	35	58
1/2010	40	80	1	11	2.5	13.75	0	0	0	0
2/2010	10	20	0	4	0	20	0	0	0	0
3/2010	40	80	0	8	0	10	0	0	0	0
4/2010	50	97	0	18	0	18.6	0	5	0	27.8
5/2010	30	60	0	2	0	3.3	0	0	0	0
6/2010	40	80	3	8	7.5	10	0	0	0	0
7/2010	40	80	2	27	5	33.8	0	10	0	37
8/2010	40	78	0	11	0	14.1	0	0	0	0
9/2010	20	40	0	8	0	20	0	0	0	0
10/2010	30	60	3	19	10	31.7	0	1	0	5.3
11/2010	40	80	2	10	5	12.5	1	0	50	0
12/2010	20	40	0	8	0	20	0	2	0	25
Total in year 2010	400	795	11	134	2.8	16.9	1	18	9	13.4
1/2011	20	40	4	9	20	22.5	4	1	100	11.1
2/2011	10	20	8	13	80	65	1	3	12.5	23.1
3/2011	20	40	3	17	15	42.5	0	1	0	5.9
4/2011	20	40	4	5	20	12.5	1	0	25	0
5/2011	20	40	1	2	5	5	1	0	100	0
6/2011	30	60	2	13	6.7	21.7	2	2	100	15.4
7/2011	10	20	0	6	0	30	0	3	0	50
8/2011	20	40	0	0	0	0	0	0	0	0
9/2011	14	39	0	1	0	2.6	0	0	0	0
10/2011	20	30	0	3	0	10	0	1	0	33.3
11/2011	20	22	0	7	0	31.8	0	0	0	0
12/2011	10	20	1	3	10	15	0	1	0	33.3
Total in year 2011	214	411	23	79	10.7	19.2	9	12	39.1	15.2
Total of three years	1187	1934	54	282	4.5	14.6	17	70	31	25

* The ratio of co-infection is number of samples vs LPAIV positive samples.

**Table 2:** Mixed infections of different of HA subtypes of LPAIV in chickens and ducks.

Type of mixed infection	Type of mixed infection	Number of case	
		Chicken	Duck
1	H1+ H3	0	1
2	H1+ H9	1	0
3	H1+ H4	0	3
4	H3+ H4	4	19
5	H3+ H6	0	3
6	H3+ H11	0	1
7	H3+ H9	0	1
8	H4+ H6	1	1
9	H4+ H9	1	4
10	H4+ H11	1	0
11	H6+ H11	0	3
12	H6+ H9	1	0
13	H9+ H11	0	1
14	H1+ H3+ H6	1	1
15	H1+ H4+ H9	2	0
16	H1+ H6+ H9	1	0
17	H3+ H4+ H6	0	2
18	H3+ H4+ H10	0	1
19	H3+ H4+ H9	0	1
20	H3+ H6+ H11	0	3
21	H4+ H6+ H9	0	1
22	H4 + H6+ H11	1	1
23	H6+ H9+ H11	1	1
24	H3+ H6+ H10+ H11	0	1
25	H3+ H6+ H9 + H11	0	4
26	H3+ H9+ H10+ H11	1	0
27	H4+ H6+ H9+ H11	0	1
28	H3+ H4+ H6 + H9+ H11	0	7
29	H3+ H6+ H9 + H10+ H11	1	9

## Discussion

With periodically emerging novel viral strains, the influenza A virus has caused devastating pandemics and has been identified as a major threat to public health, worldwide. The Guangxi province is located in southern China and is currently one of the most active areas for epidemic influenza in the world. The Guangxi province is adjacent to Vietnam, where avian influenza is also endemic and has a complicated epidemiology of human influenza.

Poultry farming is a well-developed industry in this area. There are several large-scale poultry farms, as well as a large number of small-scale farms and villages in the country. The booming poultry farming industry in this area poses a great public health risk for avian influenza. Nevertheless, epidemiological surveillance of LPAIV in this area is largely unknown. In this study, we conducted three years (2009–2011) of epidemiological surveillance of different HA subtype of LPAIV targeting the chickens and ducks sold at LBMs in the Guangxi province. Our results suggested a high prevalence of LPAIV in the poultry at these markets in southern China. We identified at least 7 HA and 5 NA subtypes of LPAIV in chickens and ducks from this area. In addition, LPAIV were isolated in all seasons, as this area has a particularly warm and humid environment, which may benefit the survival, growth and transmission of LPAIV. We also observed that the isolation rates of LPAIV in ducks were significantly higher than in chickens, which further supports the hypothesis that ducks are the major natural reservoirs of AIVs [4], [20]. It has been reported that the colonic epithelial cells in chickens express both a sialic acid α2–3 galactosidase (Siaαa2–3Gal) receptor that binds to AIVs and a sialic acid α2–6 galactosidase (Siaα2–6Gal) receptor that binds to the human influenza virus [21]–[26]. The human influenza virus receptor, the Siaα2–6Gal, is the dominant receptor. This suggests that chickens may serve as the intermediate host and thus may be the source of transmission of the influenza virus to humans [23]. Therefore, the role of the chicken in the evolution and ecology of the influenza virus needs to be investigated further.

The genome of the influenza virus contains eight RNA fragments. Mixed infections with multiple virus types could lead to reassortment [20], [27]. In addition, concomitant infections of the fungal pathogen *Cryptococcus neoformans* may be associated with enhanced virulence [28]. Genetic reassortment and recombination can occur during the process of viral proliferation and assembly in the host cells, which is a highly efficient way for AIVs to mutate and then to generate a novel virus with new phenotypes. It is thought that the natural reservoir of AIV is the wild bird population. AIVs are capable of switching hosts and causing outbreaks in new species. In nature, the high prevalence of mixed infections in chickens and ducks, as found in our study, suggest that genome reassortment may occur and result in antigenic shift. For example, the H3N2 influenza virus that caused the flu pandemic in Hong Kong in 1968 was generated by gene rearrangement of HA and PB1 from the H3 subtype of AIV [8], [11], [12]. The H5N1 subtype of avian influenza virus that caused human infection and death in Hong Kong in 1997 also originated from genetic rearrangement [29]. The HA gene from H5N1 originated from A/goose/Guangdong/1/96 (H5N1), isolated from a goose in Guangdong province in 1996 [30]. Subsequently, gene rearrangement of H5N1 with other AIVs led to the novel virus that caused the chicken avian influenza in Hong Kong in 2001 [31]. As has been previously reported in eastern China, we discovered mixed infections of HA subtypes in Guangxi. Concurrent infection was more frequent in ducks than in chickens, with concomitant infections of up to five different HA subtypes of LPAIV and 23 different kinds of mixed infections, total. Our study supported the hypothesis that ducks are the main LPAIV reservoir and promote mixed infections of different HA subtypes of LPAIV [20]. The warm, humid climate in the Guangxi province may facilitate the survival, growth and transmission of LPAIV, as well as the occurrence of mixed infections. Further investigation focusing on whether genome rearrangement occurs during mixed infection is needed.

Interestingly, the distribution and the isolation rate of the H3 subtype of LPAIV in ducks was significantly higher than other HA subtypes of LPAIV (*p*<0.01) (Figure 3). The isolation rate of the H3 subtype in chickens is also high, and is only slightly lower than the H9 subtype LPAIV (Figure 3). The H3 subtype causes the seasonal influenza widely found in humans [32], which highlights the need for further investigation and epidemiological surveillance, as well as genetic and evolutionary studies of the H3 subtypes of the influenza virus. Unlike ducks, the H9 subtype was the most commonly isolated subtype of LPAIV in chickens. The H9 subtype of LPAIV has not only caused economic losses for the poultry industry, but is also capable of infecting humans by directly crossing hosts, which poses a serious threat to human health [13], [14], [33]. It is not clear whether the H9 subtype of LPAIV mutated, or experienced antigenic drift, or whether genetic recombination occurs between the H9 subtype and the H5 subtype of LPAIV, which would improve pathogenicity.

In conclusion, we investigated the epidemiology of LPAIV in poultry from LBMs in the Guangxi province of southern China, a hotbed for the avian flu. Our study demonstrates a high prevalence of LPAIV in the poultry in this area and highlights the significant need for further investigation of the genetics and evolutionary of LPAIV.
